# Supplementary material for: EXO1 as a therapeutic target for Fanconi Anaemia, ZRSR2 and BRCA1-A complex deficient cancers
Source: Nat Commun. 2025 Sep 26;16:8476. doi: 10.1038/s41467-025-63349-7 (PMC12475092; doi:10.1038/s41467-025-63349-7)

Maric et al. Supplementary Figure 1

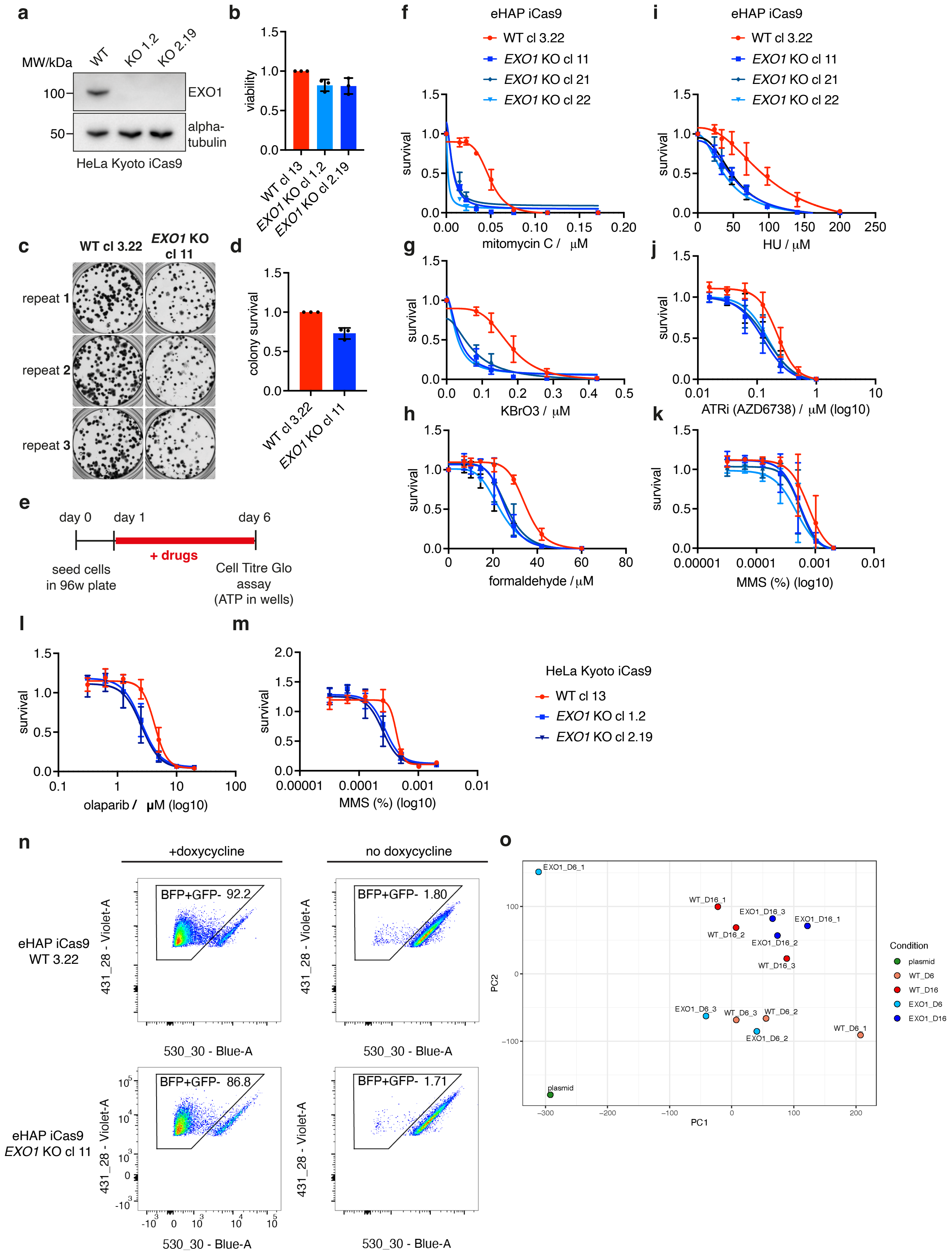

Maric et al. Supplementary Figure 1 – Validation of *EXO1* KO cell lines through their viability and sensitivity to DNA damaging agents and inhibitors of DDR factors

(a) Immunoblotting for EXO1 protein levels in HeLa Kyoto iCas9 *EXO1* KO clones (alpha-tubulin as control for equal loading; n=3).

(b) Viability of HeLa Kyoto iCas9 *EXO1* KO clones based on CellTitre-Glo assay (n=3, mean with SD); normalised against wild type levels.

(c) Representative images of clonogenic assay for eHAP iCas9 *EXO1* KO clone 11 (n=3, well diameter 15.5mm).

(d) Quantification of clonogenic assay for eHAP iCas9 *EXO1* KO clone 11 (n=3, mean with SD); normalised against wild type levels.

(e) Scheme for CellTitre-Glo assay experiments addressing sensitivity of eHAP iCas9 *EXO1* KO clones to DNA damaging agents and inhibitors of DDR factors.

(f) Sensitivity of eHAP iCas9 *EXO1* KO clones to DNA crosslinker mitomycin C (n=3, mean with SD).

(g) Sensitivity of eHAP iCas9 *EXO1* KO clones to oxidating agent potassium bromate (n=3, mean with SD).

(h) Sensitivity of eHAP iCas9 *EXO1* KO clones to DNA-protein crosslinker formaldehyde (n=3, mean with SD).

(i) Sensitivity of eHAP iCas9 *EXO1* KO clones to ribonucleotide reductase inhibitor hydroxyurea (HU) (n=3, mean with SD).

(j) Sensitivity of eHAP iCas9 *EXO1* KO clones to inhibitor of checkpoint kinase ATR (AZD6738) (n=3, mean with SD).

(k) Sensitivity of eHAP iCas9 *EXO1* KO clones to alkylating agent methyl methanesulfonate (MMS) (n=3, mean with SD).

(l) Sensitivity of HeLa Kyoto iCas9 *EXO1* KO clones to PARP inhibitor olaparib (n=3, mean with SD).

(m) Sensitivity of HeLa Kyoto iCas9 *EXO1* KO clones to MMS (n=3, mean with SD).

(n) Cas9 editing efficiency assay for eHAP iCas9 wild type and *EXO1* KO cell lines which were used for the CRISPR screen; cells not treated with doxycycline as control and readout of base levels of editing.

(o) PCA (Principal Component Analysis) of samples from genome-wide CRISPR dropout screen in eHAP iCas9 wild type and *EXO1* KO isogenic pair.

Maric et al. Supplementary Figure 2

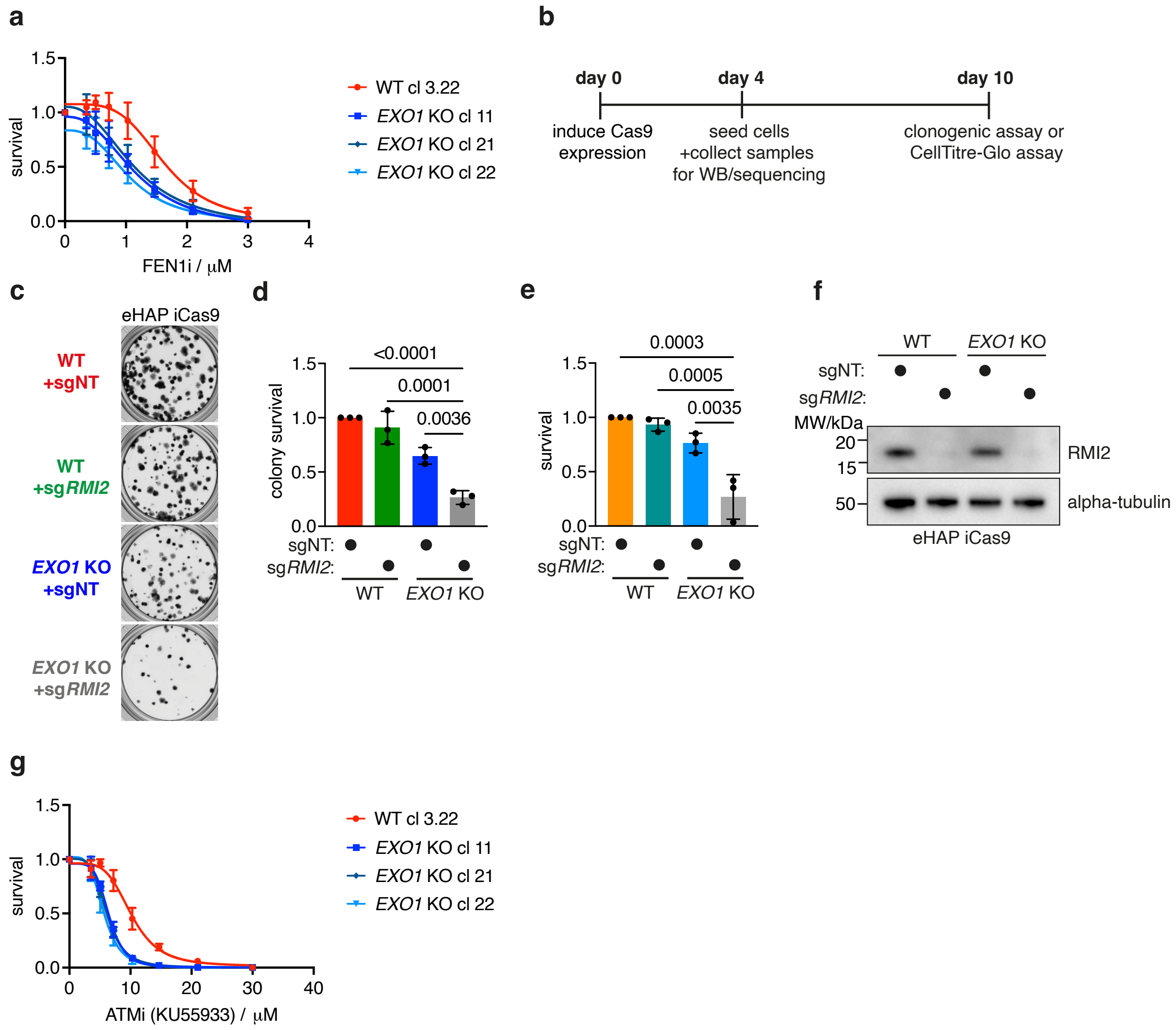

Maric et al. Supplementary Figure 2 – Validation of synthetic lethal interactions with loss of *EXO1* for genes that were previously shown to be synthetic lethal in model organisms and reciprocal screens

(a) Validation of *EXO1* and *FEN1* synthetic lethal interaction through sensitivity of eHAP iCas9 *EXO1* KO clones to FEN1 inhibitor in CellTitre-Glo assay (n=3, mean with SD).

(b) Scheme of clonogenic assay and CellTitre-Glo viability assay experiments for validation of synthetic lethal interactions identified in the genome-wide CRISPR dropout screen in eHAP iCas9 *EXO1* KO vs wild type.

(c) Validation of *EXO1* and *RMI2* synthetic lethal interaction in eHAP iCas9 cells by clonogenic assay (representative images, n=3, well diameter 15.5mm).

(d) Quantification of clonogenic assay (n=3, mean with SD) in (c) with ordinary one-way ANOVA statistical analysis (F=39.77).

(e) Validation of *EXO1* and *RMI2* synthetic lethal interaction in eHAP iCas9 cells by CellTitre-Glo viability assay (n=3, mean with SD) with ordinary one-way ANOVA statistical analysis (F=24.34).

(f) Immunoblotting for RMI2 protein levels upon induction of Cas9 expression (alpha-tubulin as control for equal loading).

(g) Validation of *EXO1* and *ATM* synthetic lethal interaction through sensitivity of eHAP iCas9 *EXO1* KO clones to ATM inhibitor (KU55933) in CellTitre-Glo assay (n=3, mean with SD).

Maric et al. Supplementary Figure 3

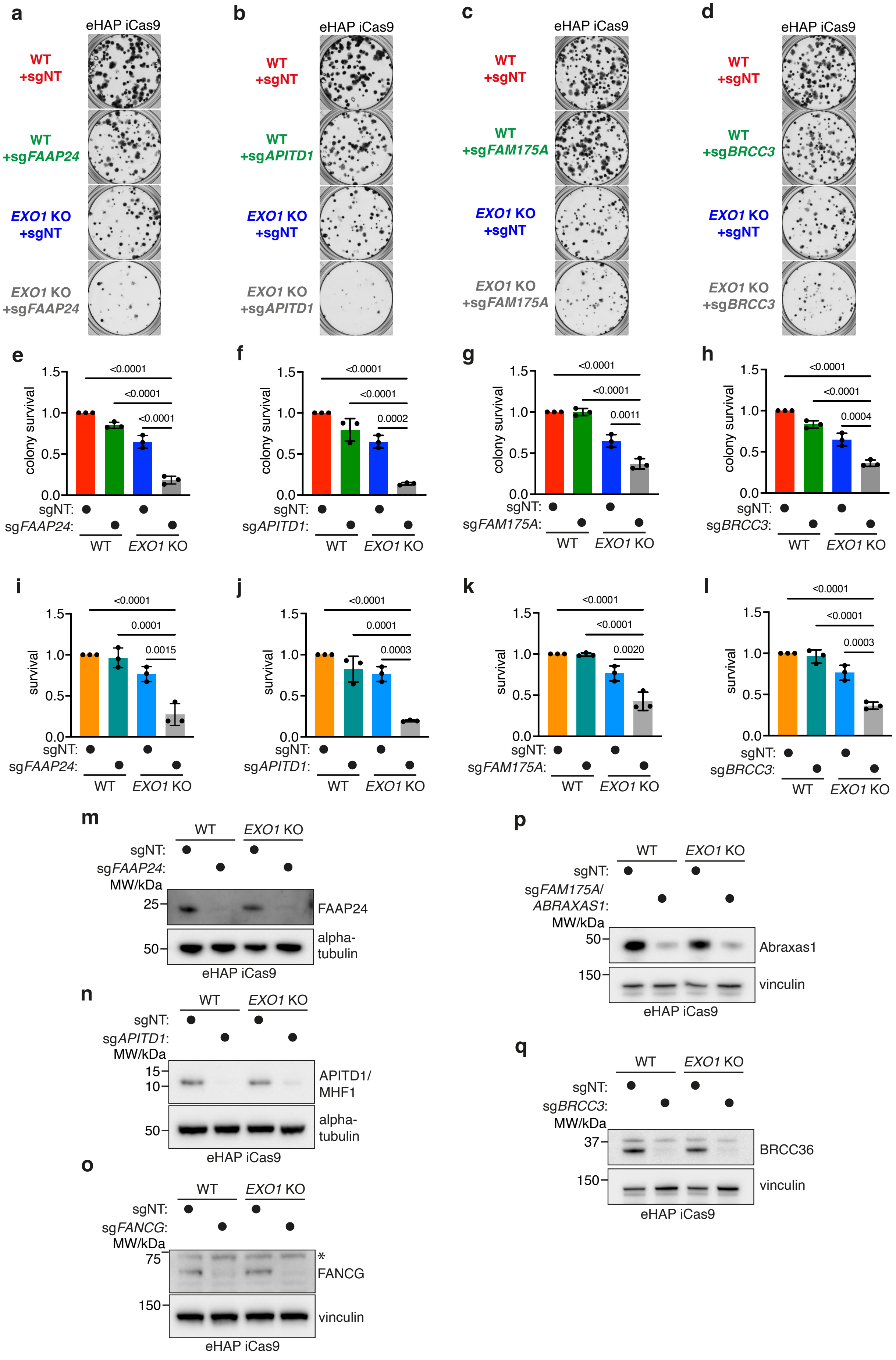

Maric et al. Supplementary Figure 3 – Validation of synthetic lethal interactions with loss of *EXO1* for DDR factors which are frequently mutated in cancers (in eHAP iCas9 cell line)

(a) Validation of *EXO1* and *FAAP24* synthetic lethal interaction in eHAP iCas9 cells by clonogenic assay (representative images, n=3, well diameter 15.5mm).

(b) Validation of *EXO1* and *APITD1/MHF1* synthetic lethal interaction in eHAP iCas9 cells by clonogenic assay (representative images, n=3, well diameter 15.5mm).

(c) Validation of *EXO1* and *FAM175A/ABRAXAS1* synthetic lethal interaction in eHAP iCas9 cells by clonogenic assay (representative images, n=3, well diameter 15.5mm).

(d) Validation of *EXO1* and *BRCC3/BRCC36* synthetic lethal interaction in eHAP iCas9 cells by clonogenic assay (representative images, n=3, well diameter 15.5mm).

(e) Quantification of clonogenic assay (n=3, mean with SD) in (a) with ordinary one-way ANOVA statistical analysis (F=158.2).

(f) Quantification of clonogenic assay (n=3, mean with SD) in (b) with ordinary one-way ANOVA statistical analysis (F=65.50).

(g) Quantification of clonogenic assay (n=3, mean with SD) in (c) with ordinary one-way ANOVA statistical analysis (F=94.86).

(h) Quantification of clonogenic assay (n=3, mean with SD) in (d) with ordinary one-way ANOVA statistical analysis (F=94.90).

(i) Validation of *EXO1* and *FAAP24* synthetic lethal interaction in eHAP iCas9 cells by CellTitre-Glo viability assay (n=3, mean with SD) with ordinary one-way ANOVA statistical analysis (F=33.23).

(j) Validation of *EXO1* and *APITD1/MHF1* synthetic lethal interaction in eHAP iCas9 cells by CellTitre-Glo viability assay (n=3, mean with SD) with ordinary one-way ANOVA statistical analysis (F=44.08).

(k) Validation of *EXO1* and *FAM175A/ABRAXAS1* synthetic lethal interaction in eHAP iCas9 cells by CellTitre-Glo viability assay (n=3, mean with SD) with ordinary one-way ANOVA statistical analysis (F=41.43).

(l) Validation of *EXO1* and *BRCC3/BRCC36* synthetic lethal interaction in eHAP iCas9 cells by CellTitre-Glo viability assay (n=3, mean with SD) with ordinary one-way ANOVA statistical analysis (F=61.37).

(m) Immunoblotting for FAAP24 protein levels upon induction of Cas9 expression (alpha-tubulin as equal loading control).

(n) Immunoblotting for APITD1/MHF1 protein levels upon induction of Cas9 expression (alpha-tubulin as equal loading control).

(o) Immunoblotting for FANCG protein levels upon induction of Cas9 expression (vinculin as equal loading control).

(p) Immunoblotting for Abraxas1 protein levels upon induction of Cas9 expression (vinculin as equal loading control).

(q) Immunoblotting for BRCC36 protein levels upon induction of Cas9 expression (vinculin as equal loading control).

Maric et al. Supplementary Figure 4

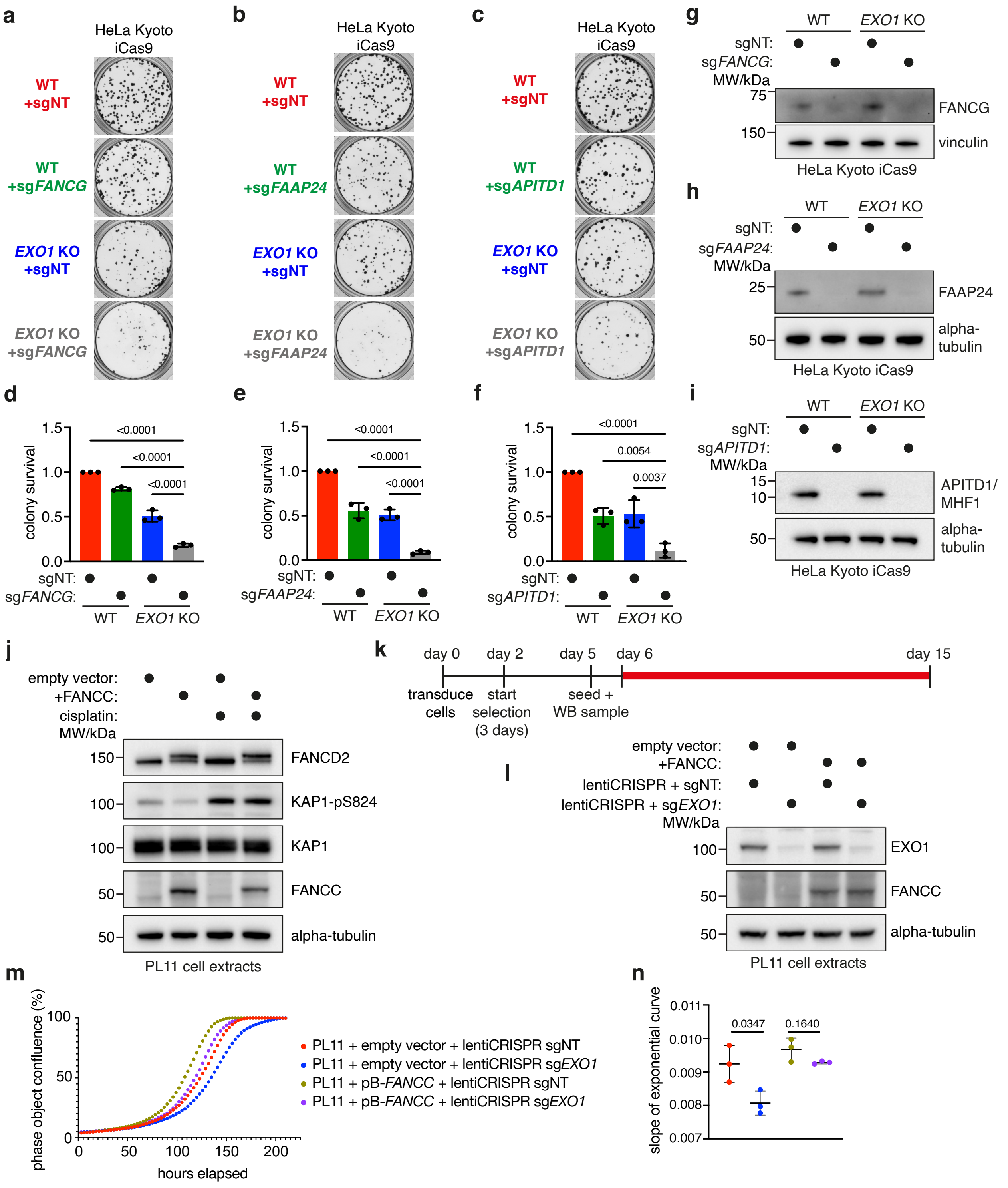

Maric et al. Supplementary Figure 4 – Validation of synthetic lethal interactions with loss of *EXO1* for FA genes in HeLa Kyoto iCas9 cell line and PL11 cancer cell line

(a) Validation of *EXO1* and *FANCG* synthetic lethal interaction in HeLa Kyoto iCas9 cells by clonogenic assay (representative images, n=3, well diameter 15.5mm). HeLa Kyoto iCas9 *EXO1* KO clone 2.19 was used as the parental cell line for *EXO1* KO.

(b) Validation of *EXO1* and *FAAP24* synthetic lethal interaction in HeLa Kyoto iCas9 cells (clone 2.19) by clonogenic assay (representative images, n=3, well diameter 15.5mm).

(c) Validation of *EXO1* and *APITD1/MHF1* synthetic lethal interaction in HeLa Kyoto iCas9 cells (clone 2.19) by clonogenic assay (representative images, n=3, well diameter 15.5mm).

(d) Quantification of clonogenic assay (n=3, mean with SD) in (a) with ordinary one-way ANOVA statistical analysis (F=335.7).

(e) Quantification of clonogenic assay (n=3, mean with SD) in (b) with ordinary one-way ANOVA statistical analysis (F=141.7).

(f) Quantification of clonogenic assay (n=3, mean with SD) in (c) with ordinary one-way ANOVA statistical analysis (F=41.15).

(g) Immunoblotting for FANCG protein levels upon induction of Cas9 expression (vinculin as control for equal loading).

(h) Immunoblotting for FAAP24 protein levels upon induction of Cas9 expression (alpha-tubulin as control for equal loading).

(i) Immunoblotting for APITD1/MHF1 protein levels upon induction of Cas9 expression (alpha-tubulin as control for equal loading).

(j) Immunoblotting analysis of total cell extracts for PL11 + empty vector and PL11 + FANCC without and with cisplatin treatment (100μM for 2 hours). Samples were probed for FANCD2, phosphorylated levels of KAP1 (pS824) with total KAP1 as control, FANCC, as well as alpha-tubulin as control for equal loading.

(k) Scheme of experiments analysing cell proliferation with Incucyte for validation of the effect of *EXO1* knockout on FANCC-deficient and FANCC-expressing cancer cell line PL11 (WB – Western Blot).

(l) Immunoblotting of a representative biological repeat of PL11 transductions for EXO1 and FANCC protein levels (alpha-tubulin as control for equal loading).

(m) Representative experiment for cell proliferation assay for PL11 + empty vector + lentiCRISPR sgNT, PL11 + empty vector + lentiCRISPR sg*EXO1*, PL11 + piggyBac-FANCC + lentiCRISPR sgNT, PL11 + piggyBac-FANCC + lentiCRISPR sg*EXO1* (n=3).

(n) Slope of exponential curve fit for transduced PL11 cells (n=3, mean with SD) with two-tailed paired t-test statistical analysis.

Maric et al. Supplementary Figure 5

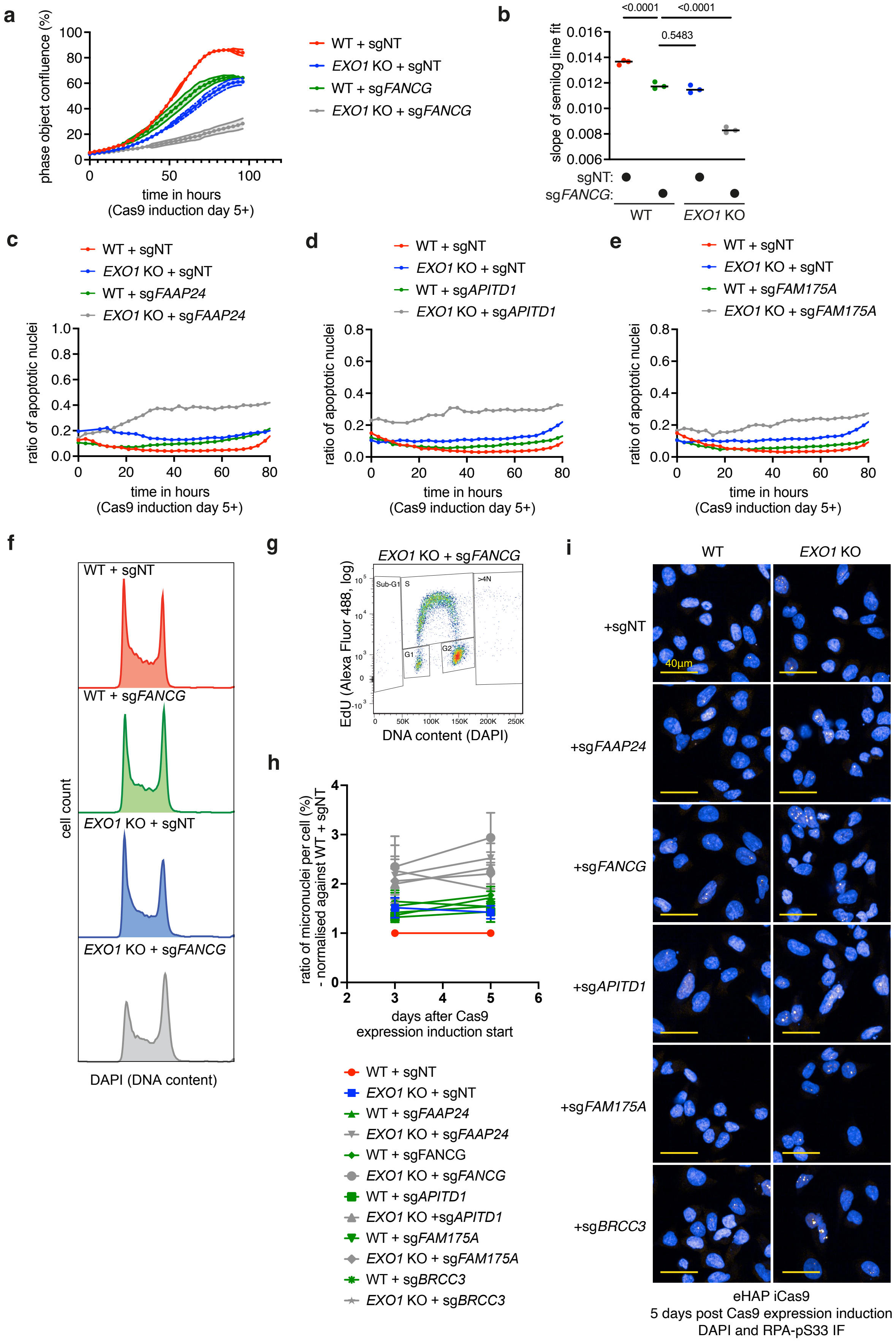

Maric et al. Supplementary Figure 5 – Genome instability, cell cycle arrest in G2 phase and apoptosis induced by FA pathway gene and BRCA1-A complex gene knockouts in *EXO1* KO cells

(a) Cell proliferation assay for 'WT + sgNT', 'WT + sg*FANCG*', '*EXO1* KO + sgNT' and '*EXO1* KO + sg*FANCG*' eHAP iCas9 cells (representative biological repeat, n=3).

(b) Plot of slope of semilog line fits for cell proliferation curves as shown in (a) (n=3, line representing mean, ordinary one-way ANOVA analysis, F=238.4).

(c) Apoptosis of *EXO1* and *FAAP24* double KO cells (n=3, representative biological repeat).

(d) Apoptosis of *EXO1* and *APITD1* double KO cells (n=3, representative biological repeat).

(e) Apoptosis of *EXO1* and *FAM175A* double KO cells (n=3, representative biological repeat).

(f) Representative overlay for histogram plot of a cell cycle analysis for 'WT + sgNT', 'WT + sg*FANCG*', '*EXO1* KO + sgNT' and '*EXO1* KO + sg*FANCG*' cell lines (n=4).

(g) G2 cell cycle arrest of double KO *EXO1-FANCG* cells (representative biological repeat, n=4).

(h) Timecourse of micronuclei increase in double KO cells (day 5 of the timecourse shown in Fig.2f). For each cell line (nuclei number > 2182, n=3) ratio was calculated by normalisation to 'WT + sgNT' cell line.

(i) Representative images of genome instability phenotypes of double KO cell lines for *EXO1* loss with *FAAP24*, *FANCG*, *APITD1*, *FAM175A* and *BRCC3* (day 5 post

Cas9 expression induction; DAPI staining and RPA-pS33 immunofluorescence staining; n=3). Scale bar: 40μm.

Maric et al. Supplementary Figure 6

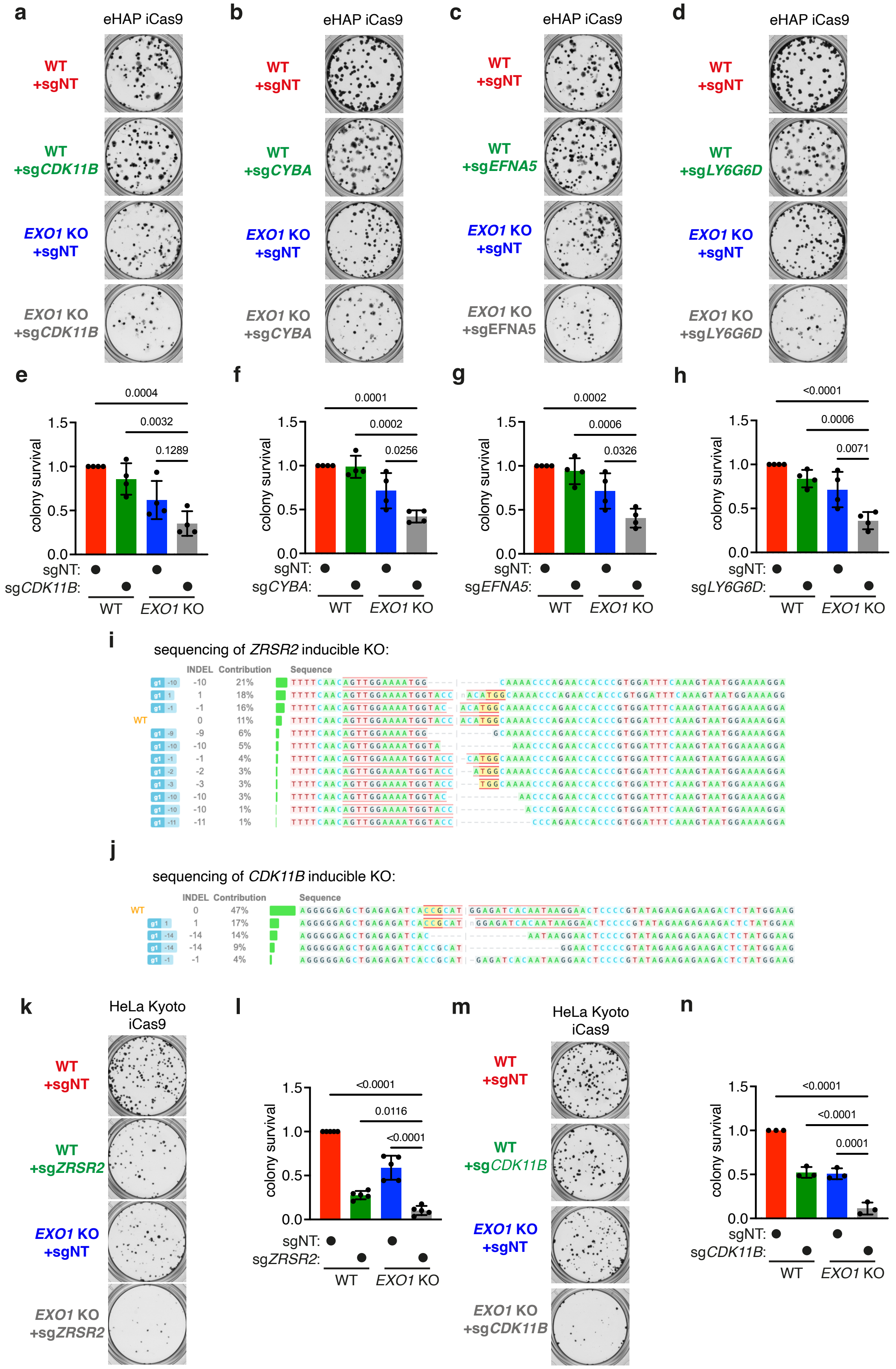

Maric et al. Supplementary Figure 6 – Validation of synthetic lethal interactions with loss of *EXO1* for *CDK11B*, *CYBA*, *EFNA5* and *LY6G6D* in eHAP iCas9 cell line and for *ZRSR2* and *CDK11B* in HeLa Kyoto iCas9 cell line

(a) Validation of *EXO1* and *CDK11B* synthetic lethal interaction in eHAP iCas9 cells by clonogenic assay (representative images, n=4, well diameter 15.5mm).

(b) Validation of *EXO1* and *CYBA* synthetic lethal interaction in eHAP iCas9 cells by clonogenic assay (representative images, n=4, well diameter 15.5mm).

(c) Validation of *EXO1* and *EFNA5* synthetic lethal interaction in eHAP iCas9 cells by clonogenic assay (representative images, n=4, well diameter 15.5mm).

(d) Validation of *EXO1* and *LY6G6D* synthetic lethal interaction in eHAP iCas9 cells by clonogenic assay (representative images, n=4, well diameter 15.5mm).

(e) Quantification of clonogenic assay (n=4, mean with SD) in (a) with ordinary one-way ANOVA statistical analysis (F=13.11).

(f) Quantification of clonogenic assay (n=4, mean with SD) in (b) with ordinary one-way ANOVA statistical analysis (F=19.55).

(g) Quantification of clonogenic assay (n=4, mean with SD) in (c) with ordinary one-way ANOVA statistical analysis (F=15.69).

(h) Quantification of clonogenic assay (n=4, mean with SD) in (d) with ordinary one-way ANOVA statistical analysis (F=19.75).

(i) Deconvolved sequencing result for inducible KO of *ZRSR2* (representative example).

(j) Deconvolved sequencing result for inducible KO of *CDK11B* (representative example).

(k) Validation of *EXO1* and *ZRSR2* synthetic lethal interaction in HeLa Kyoto iCas9 cells (clone 2.19) by clonogenic assay (representative images, n=5, well diameter 15.5mm).

(l) Quantification of clonogenic assay (n=5, mean with SD) in (k) with ordinary one-way ANOVA statistical analysis (F=131.4).

(m) Validation of *EXO1* and *CDK11B* synthetic lethal interaction in HeLa Kyoto iCas9 cells (clone 2.19) by clonogenic assay (representative images, n=3, well diameter 15.5mm).

(n) Quantification of clonogenic assay (n=3, mean with SD) in (m) with ordinary one-way ANOVA statistical analysis (F=129.9).

Maric et al. Supplementary Figure 7

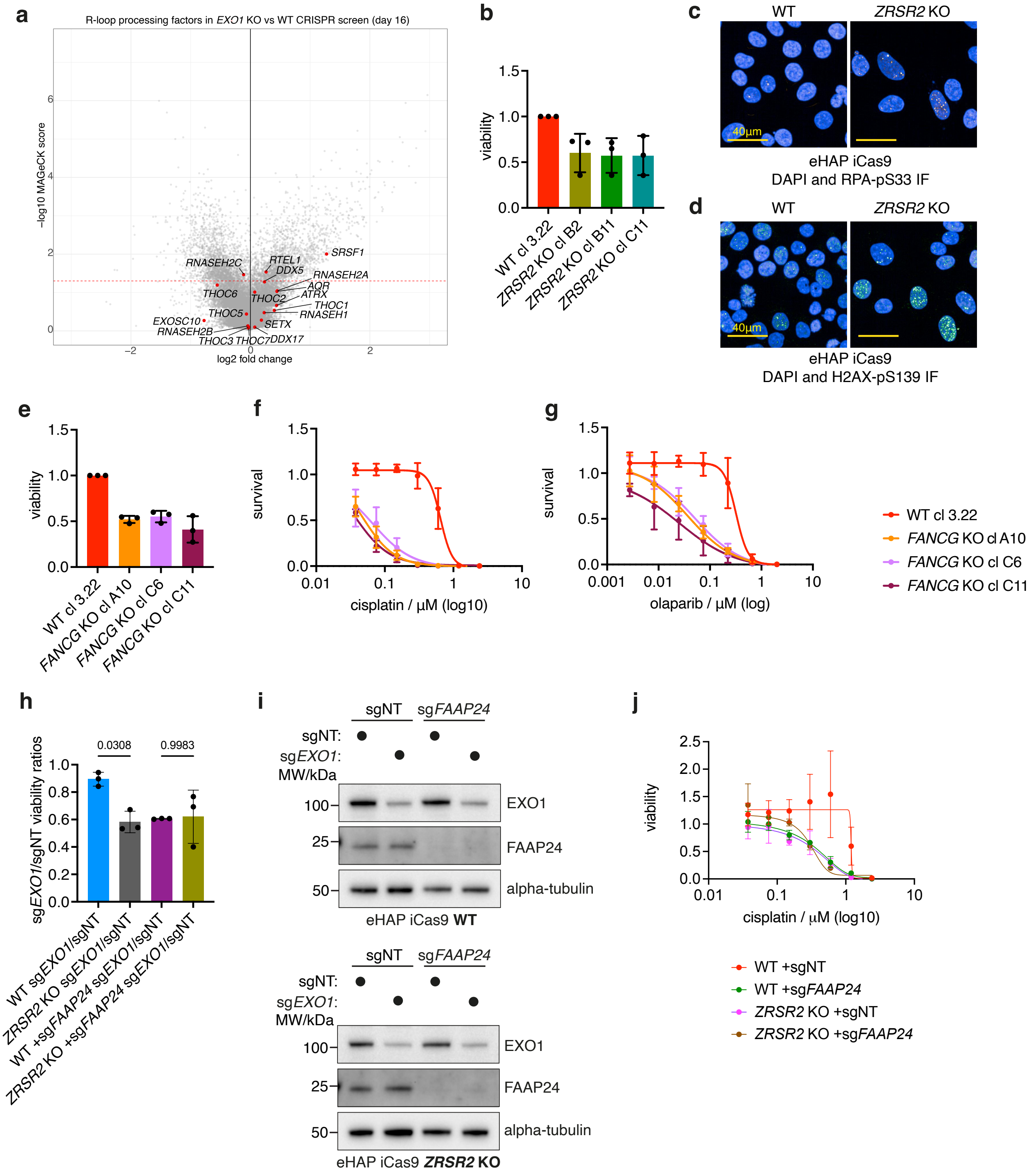

Maric et al. Supplementary Figure 7 – R-loop processing factors in *EXO1* KO vs WT CRISPR screen comparison, viability of constitutive KO clones for *ZRSR2* and *FANCG* in eHAP iCas9 background, genome instability in *ZRSR2* KO, functional validation for *FANCG* KO clones and epistasis experiments

(a) Volcano plot of the CRISPR screen 'day 16' comparison of *EXO1* KO vs WT cells with highlighted R-loop processing factors.

(b) Viability of eHAP iCas9 *ZRSR2* KO clones based on CellTitre-Glo assay (n=3, (n=4, mean with SD, normalised against wild type).

(c) Representative images for increase in RPA-pS33 foci of *ZRSR2* KO in comparison to wild type (n=3). Scale bar: 40μm.

(d) Representative images for increase in H2AX-pS139 signal intensity of *ZRSR2* KO in comparison to wild type (n=3). Scale bar: 40μm.

(e) Viability of eHAP iCas9 *FANCG* KO clones based on CellTitre-Glo assay (n=3, (n=4, mean with SD, normalised against wild type).

(f) Sensitivity of eHAP iCas9 *FANCG* KO clones to cisplatin (n=3, mean with SD).

(g) Sensitivity of eHAP iCas9 *FANCG* KO clones to olaparib (n=3, mean with SD).

(h) Ratios of viability derived from clonogenic assays between 'sgEXO1' and 'sgNT' cell lines (n=3; mean with SD, ordinary one-way ANOVA, F=5.558).

(i) Immunoblotting for EXO1 and FAAP24 protein levels upon induction of Cas9 expression in epistasis experiment in eHAP iCas9 WT and *ZRSR2* KO cells (alpha-tubulin as control for equal loading).

(j) Sensitivity of 'WT + sgNT', 'WT + sg*FAAP24*', '*ZRSR2* KO + sgNT' and '*ZRSR2* KO + sg*FAAP24*' to cisplatin (n=3, mean with SD).

Maric et al. Supplementary Figure 8

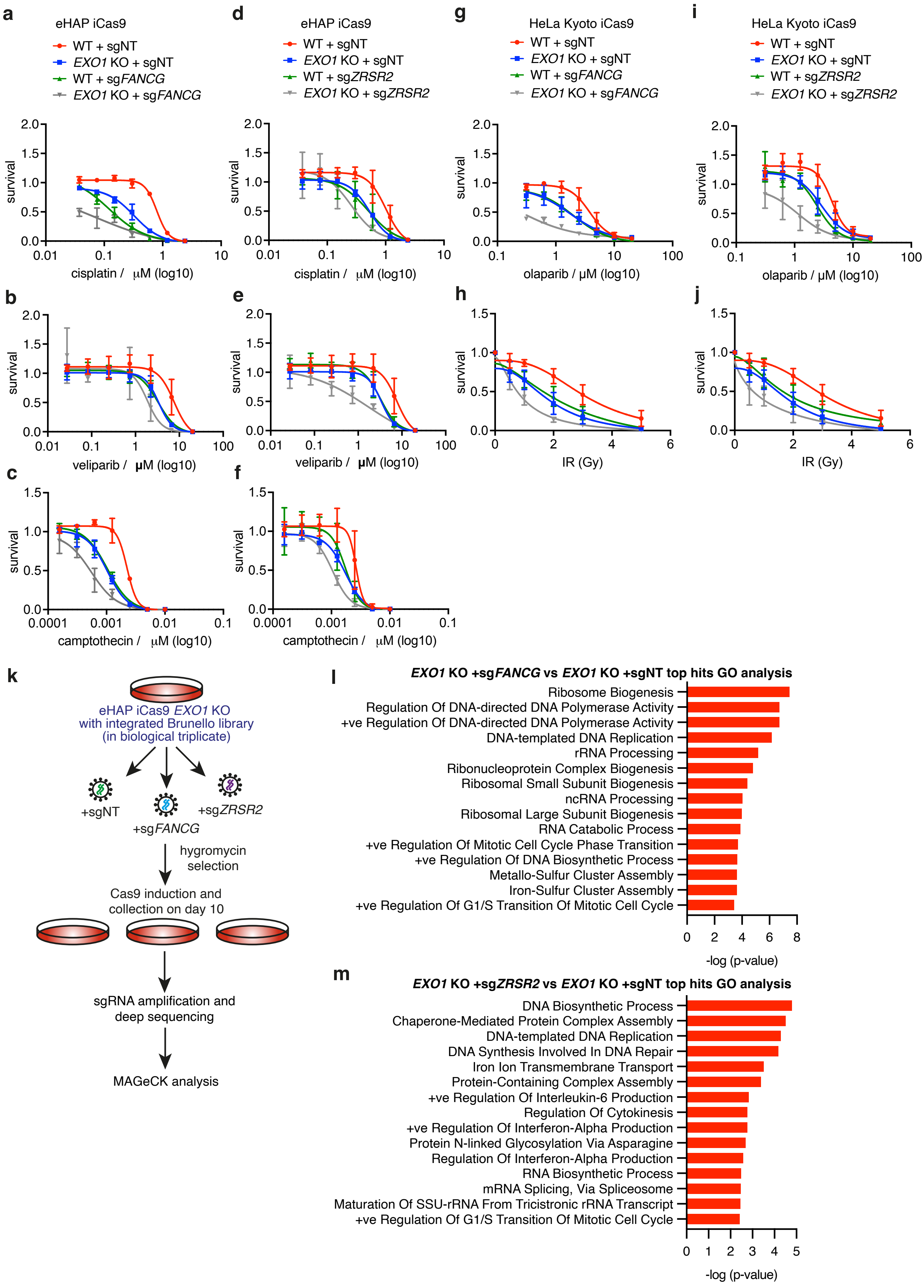

Maric et al. Supplementary Figure 8 – Additive sensitivities of cell lines with loss of *EXO1* and *FANCG* or *EXO1* and *ZRSR2*

(a) Additive sensitivity of eHAP iCas9 double KO cells for *EXO1* and *FANCG* to cisplatin (n=3, mean with SD).

(b) Additive sensitivity of eHAP iCas9 double KO cells for *EXO1* and *FANCG* to veliparib (n=3, mean with SD).

(c) Additive sensitivity of eHAP iCas9 double KO cells for *EXO1* and *FANCG* to camptothecin (n=3, mean with SD).

(d) Additive sensitivity of eHAP iCas9 double KO cells for *EXO1* and *ZRSR2* to cisplatin (n=3, mean with SD).

(e) Additive sensitivity of eHAP iCas9 double KO cells for *EXO1* and *ZRSR2* to veliparib (n=3, mean with SD).

(f) Additive sensitivity of eHAP iCas9 double KO cells for *EXO1* and *ZRSR2* to camptothecin (n=3, mean with SD).

(g) Additive sensitivity of HeLa Kyoto iCas9 double KO cells for *EXO1* and *FANCG* to olaparib (n=3, mean with SD).

(h) Additive sensitivity of HeLa Kyoto iCas9 double KO cells for *EXO1* and *FANCG* to ionising radiation (IR) (n=3, mean with SD).

(i) Additive sensitivity of HeLa Kyoto iCas9 double KO cells for *EXO1* and *ZRSR2* to olaparib (n=3, mean with SD).

(j) Additive sensitivity of HeLa Kyoto iCas9 double KO cells for *EXO1* and *ZRSR2* to ionising radiation (n=3, mean with SD).

(k) Scheme of genome-wide CRISPR rescue screen in eHAP iCas9 *EXO1* KO cells previously transduced with Brunello library (n=3), followed by transductions with

lentiviruses with sgNT or sg*FANCG* or sg*ZRSR2*, respectively. Samples were collected 10 days after Cas9 expression induction.

(l) GO Biological Process (2023) term analysis for top rescue hits from genome-wide CRISPR rescue screen in eHAP iCas9 *EXO1* KO cells (sg*FANCG* vs sgNT comparison; hits above log fold change of 2.25). P value was calculated with Fisher's exact test.

(m) GO Biological Process (2023) term analysis for top rescue hits from genome-wide CRISPR rescue screen in eHAP iCas9 *EXO1* KO cells (sg*ZRSR2* vs sgNT comparison; hits above log fold change of 1.75). P value was calculated with Fisher's exact test.

Maric et al. Supplementary Figure 9

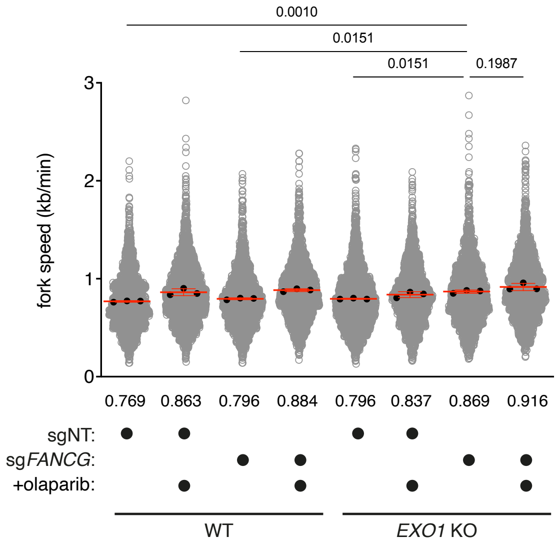

Maric et al. Supplementary Figure 9 – Treatment with high concentration of PARP inhibitor olaparib can further drive replication fork speed increase in *EXO1-FANCG* double KO cells, likely contributing to phenotype of additive sensitivity

Quantification of replication fork speed from DNA fibers experiment without and with PARP inhibitor olaparib. Measurements of a minimum of 300 fibers per condition per biological repeat (n =3). Black points represent mean from each biological repeat, with red lines representing the mean with standard deviation of those. Means are also noted below X-axis in the numerical value. Two-way ANOVA statistical analysis was performed on the means of biological repeats.

Uncropped and unprocessed scans - Maric et al. Supplementary Figure 1a

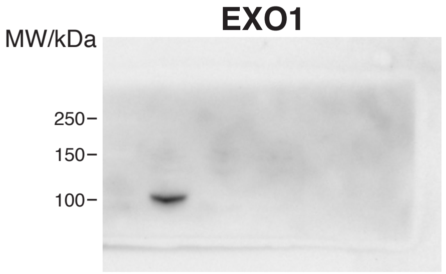

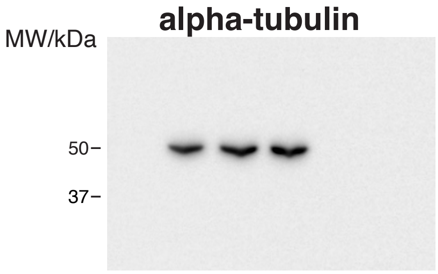

Uncropped and unprocessed scans - Maric et al. Supplementary Figure 2f

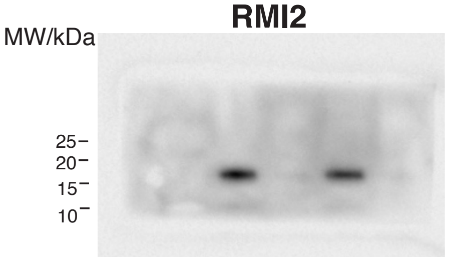

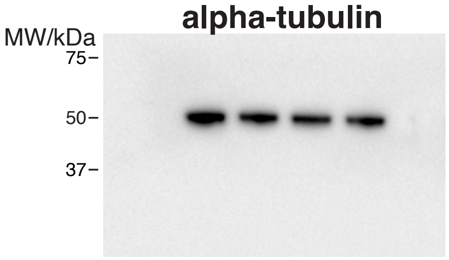

Uncropped and unprocessed scans - Maric et al. Supplementary Figure 3m

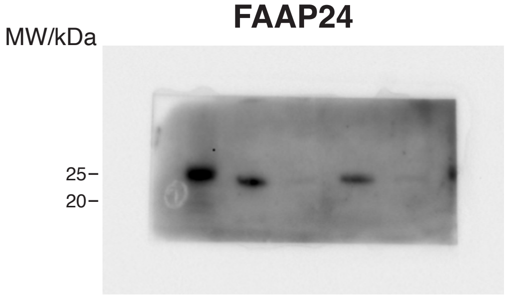

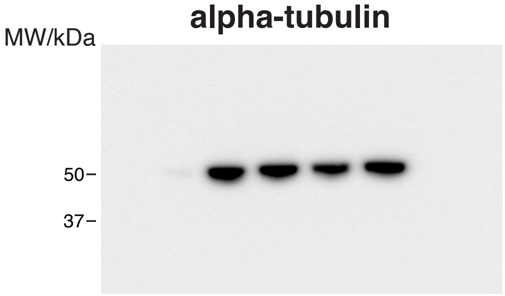

Uncropped and unprocessed scans - Maric et al. Supplementary Figure 3n

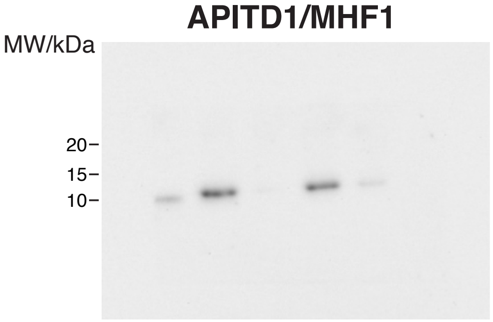

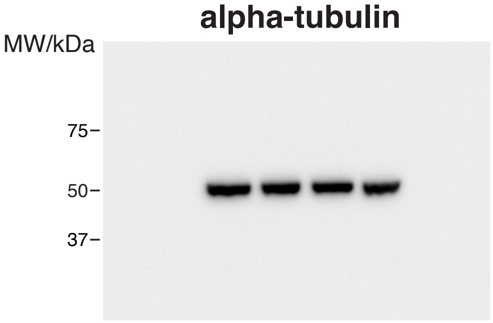

Uncropped and unprocessed scans - Maric et al. Supplementary Figure 3o

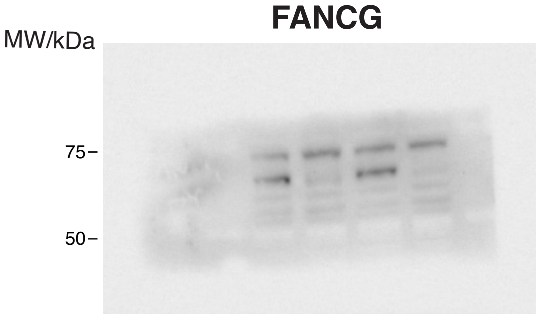

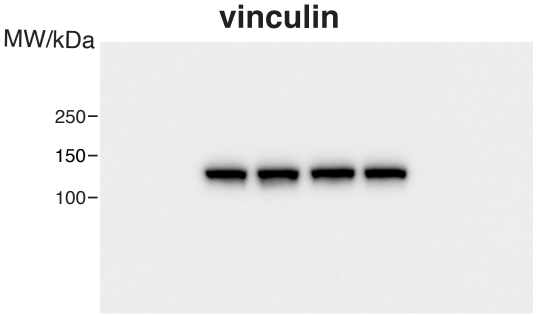

Uncropped and unprocessed scans - Maric et al. Supplementary Figure 3p

Abraxas1

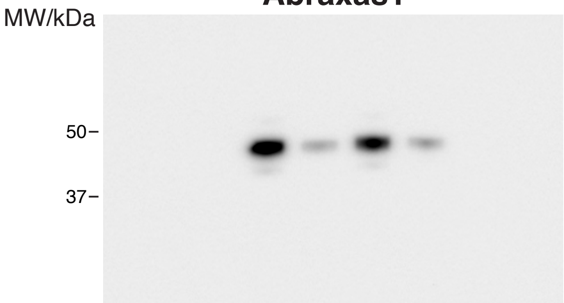

vinculin

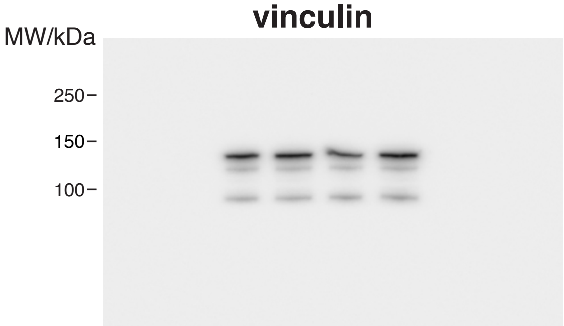

Uncropped and unprocessed scans - Maric et al. Supplementary Figure 3q

BRCC36

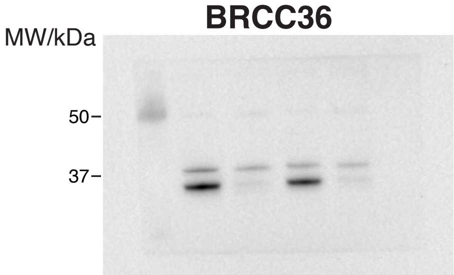

vinculin

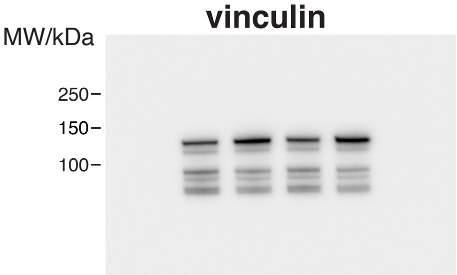

Uncropped and unprocessed scans - Maric et al. Supplementary Figure 4g

FANCG

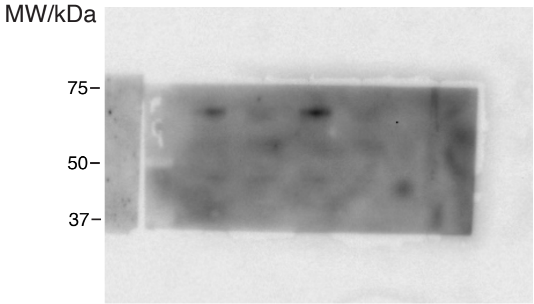

vinculin

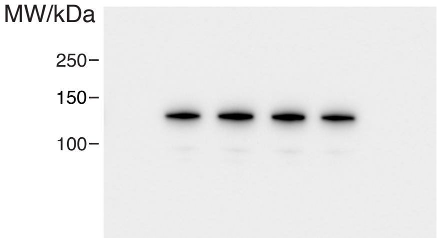

Uncropped and unprocessed scans - Maric et al. Supplementary Figure 4h

FAAP24

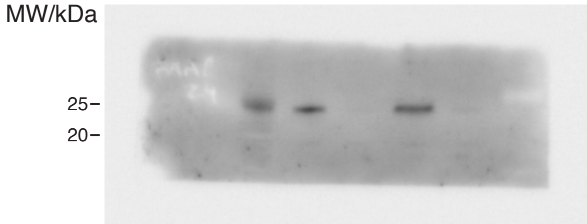

alpha-tubulin

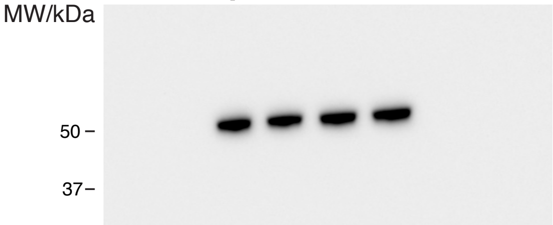

Uncropped and unprocessed scans - Maric et al. Supplementary Figure 4i

APITD1/MHF1

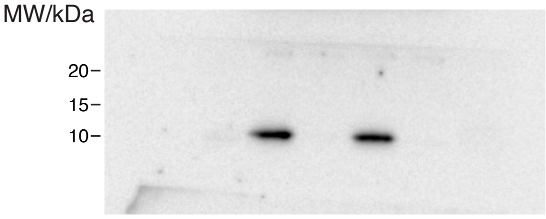

alpha-tubulin

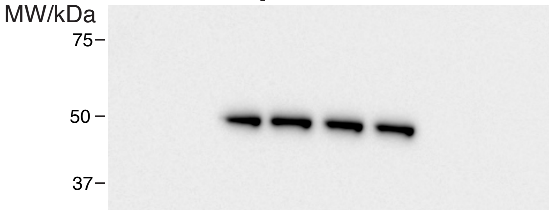

Uncropped and unprocessed scans - Maric et al. Supplementary Figure 4j

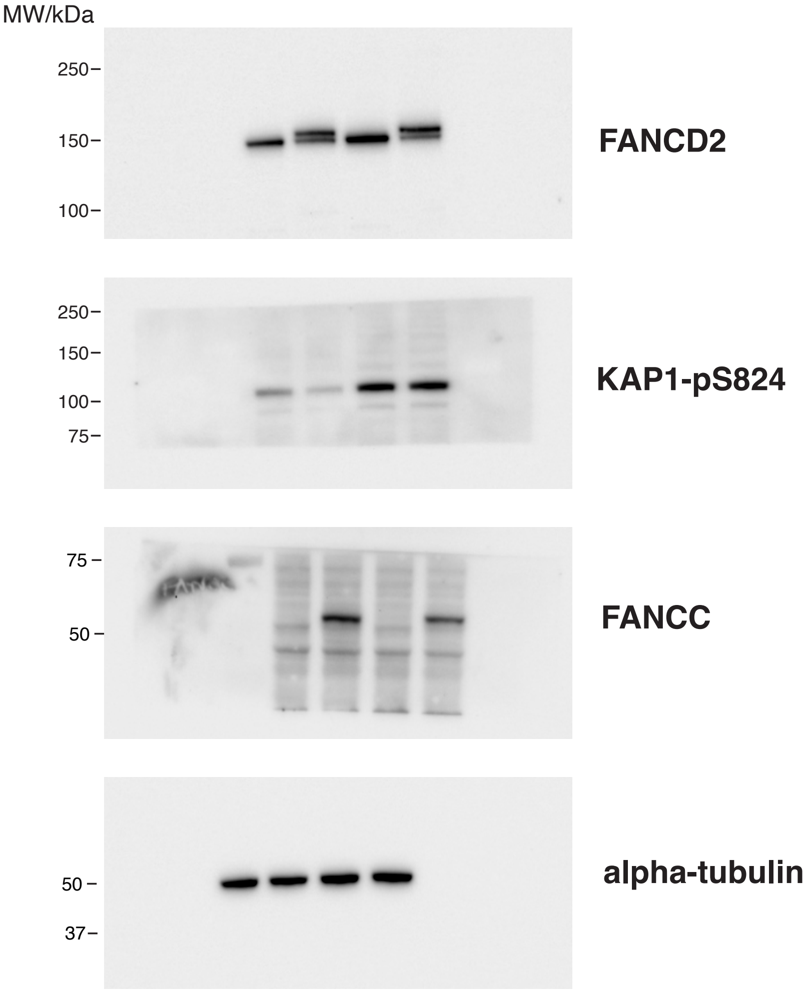

Uncropped and unprocessed scans - Maric et al. Supplementary Figure 4l

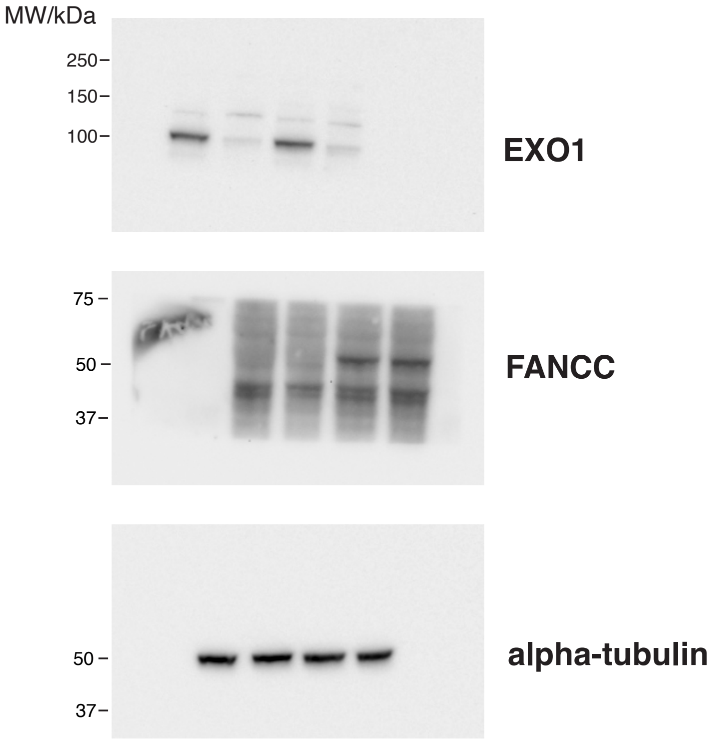

Uncropped and unprocessed scans - Maric et al. Supplementary Figure 7i

WT cells panels:

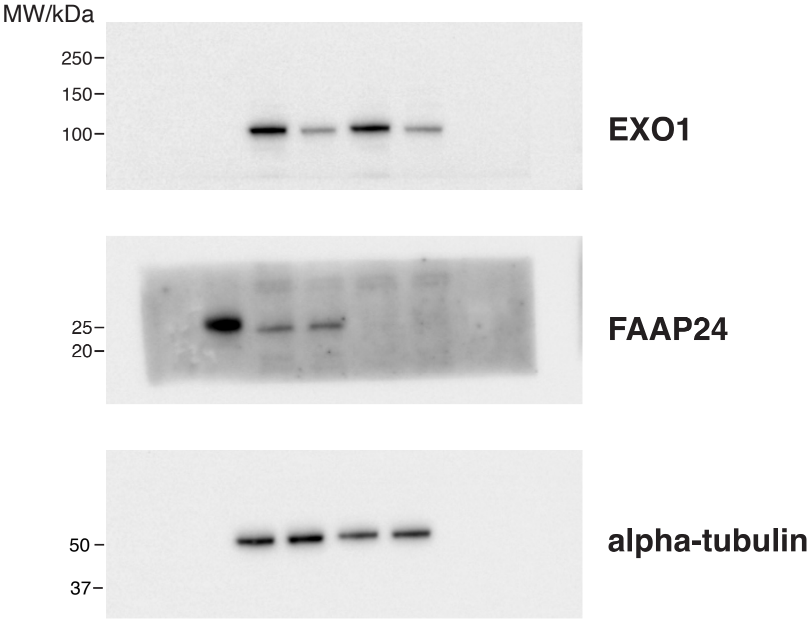

ZRSR2 KO cells panels:

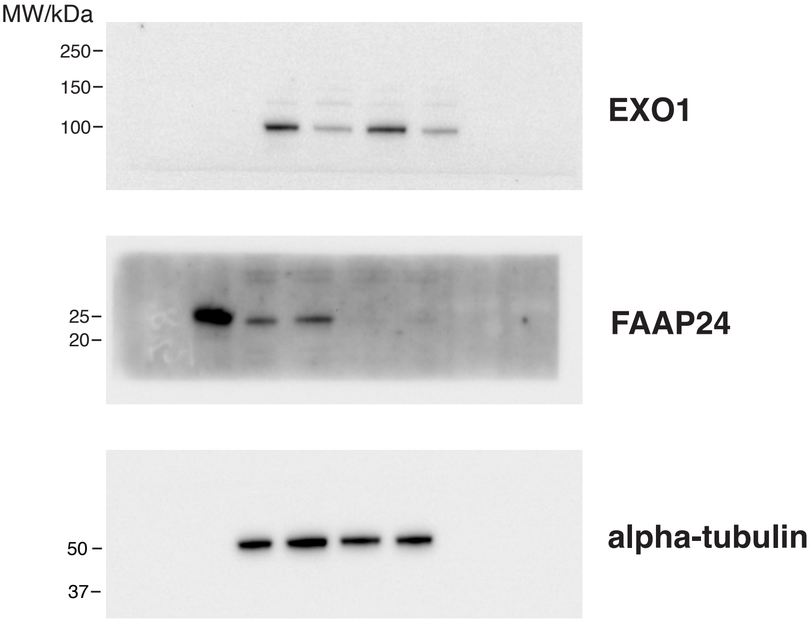

Supplement: Supplementary file 1 — Supplementary Information [file 41467_2025_63349_MOESM1_ESM.pdf]
