# Supplementary material for: EXO1 as a therapeutic target for Fanconi Anaemia, ZRSR2 and BRCA1-A complex deficient cancers
Source: Nat Commun. 2025 Sep 26;16:8476. doi: 10.1038/s41467-025-63349-7 (PMC12475092; doi:10.1038/s41467-025-63349-7)
Supplement: Supplementary file 3 — Description of Additional Supplementary Files [file 41467_2025_63349_MOESM3_ESM.pdf]

# <u>Description of Additional Supplementary Files</u>

**Supplementary Data 1 –** Genome-wide CRISPR-Cas9 screen in eHAP iCas9 wild type vs *EXO1* KO cells (n=3). MAGeCK analysis of sequencing from samples from day 6. Negative binomial model integrated in MAGeCK algorithm was used to calculate P values.

**Supplementary Data 2 –** Genome-wide CRISPR-Cas9 screen in eHAP iCas9 wild type vs *EXO1* KO cells (n=3). MAGeCK analysis of sequencing from samples from day 16. Negative binomial model integrated in MAGeCK algorithm was used to calculate P values.

**Supplementary Data 3** – BLISS score analysis for validated synthetic lethal interactions from *EXO1* KO vs WT CRISPR dropout screen. Unpaired t-test was used for the statistical analysis of actual vs theoretical values (two-tailed P values).

**Supplementary Data 4 –** Rescue screen in eHAP iCas9 *EXO1* KO cells for synthetic lethal interaction with *FANCG* (n=3). MAGeCK analysis comparisons between '*EXO1* KO + sgNT' vs '*EXO1* KO +sg*FANCG*'. Negative binomial model integrated in MAGeCK algorithm was used to calculate P values.

**Supplementary Data 5 –** Rescue screen in eHAP iCas9 *EXO1* KO cells for synthetic lethal interaction with *ZRSR2* (n=3). MAGeCK analysis comparisons between '*EXO1* KO + sgNT' vs '*EXO1* KO +sg*ZRSR2*'. Negative binomial model integrated in MAGeCK algorithm was used to calculate P values.

**Supplementary Data 6** – sgRNA target sequences used in this study.

**Supplementary Data 7 –** Oligos for cloning and sequencing used in this study.

**Supplementary Data 8** – Oligos used for amplification of integrated sgRNAs in CRISPR screening.
