# Supplementary material for: EXO1 as a therapeutic target for Fanconi Anaemia, ZRSR2 and BRCA1-A complex deficient cancers
Source: Nat Commun. 2025 Sep 26;16:8476. doi: 10.1038/s41467-025-63349-7 (PMC12475092; doi:10.1038/s41467-025-63349-7)
Supplement: Supplementary file 12 — Reporting Summary [file 41467_2025_63349_MOESM12_ESM.pdf]

nature portfolio

Corresponding author(s): Simon Boulton

Last updated by author(s): Jul 28, 2025

Reporting Summary

Nature Portfolio wishes to improve the reproducibility of the work that we publish. This form provides structure for consistency and transparency in reporting. For further information on Nature Portfolio policies, see our Editorial Policies and the Editorial Policy Checklist.

Statistics

For all statistical analyses, confirm that the following items are present in the figure legend, table legend, main text, or Methods section.

n/a	Confirmed	
☐	☒	The exact sample size (*n*) for each experimental group/condition, given as a discrete number and unit of measurement
☐	☒	A statement on whether measurements were taken from distinct samples or whether the same sample was measured repeatedly
☐	☒	The statistical test(s) used AND whether they are one- or two-sided<br>*Only common tests should be described solely by name; describe more complex techniques in the Methods section.*
☐	☒	A description of all covariates tested
☐	☒	A description of any assumptions or corrections, such as tests of normality and adjustment for multiple comparisons
☐	☒	A full description of the statistical parameters including central tendency (e.g. means) or other basic estimates (e.g. regression coefficient) AND variation (e.g. standard deviation) or associated estimates of uncertainty (e.g. confidence intervals)
☐	☒	For null hypothesis testing, the test statistic (e.g. *F*, *t*, *r*) with confidence intervals, effect sizes, degrees of freedom and *P* value noted<br>*Give P values as exact values whenever suitable.*
☒	☐	For Bayesian analysis, information on the choice of priors and Markov chain Monte Carlo settings
☒	☐	For hierarchical and complex designs, identification of the appropriate level for tests and full reporting of outcomes
☒	☐	Estimates of effect sizes (e.g. Cohen's *d*, Pearson's *r*), indicating how they were calculated

Our web collection on statistics for biologists contains articles on many of the points above.

Software and code

Policy information about availability of computer code

Data collection	For clonogenic assay analysis GelCount software was used for colony number analysis. For CellTitre-Glo assays CLARIOstar Plus Microplate Reader and its MARS software were used for luminescence signal acquisition. Cell proliferation analyses using Incucyte ZOOM System required Incucyte Basic Analyzer software for analyses of acquired images. For flow cytometry experiments BD FACSDiva software was used for signal acquisition. Microscopy data acquisition via Opera Phenix system required Harmony software. Images for metaphase spreads and DNA fibres were obtained using Nikon NIS-Elements software. EM images were acquired with MAPS software. Immunoblotting images were acquired as TIFFs using Bio-Rad ChemiDoc MP software.
Data analysis	For flow cytometry experiments FlowJo software was used for gating analysis. MAGeCK algorithm was used for analyses of sequencing files from CRISPR screens, which was visualised using R (ggplot2). For Gene Ontology Biological Process Terms analysis gene lists were processed through Enrichr portal and analyses were visualised with GraphPad Prism. Cancer genomic data was extracted via cBioPortal and processed through Microsoft Excel to generate the summary. Sequencing data that required deconvolution was analysed through Synthego ICE Analysis tool and Decodr. Cell proliferation analyses with apoptosis-specific dye required, as described in Methods, processing using UNet-based model and Cellpose to derive total nuclei numbers from confluency data. Harmony software was used for high-content microscopy data analysis. Data from clonogenic assays, viability and sensitivity assays using CellTitre-Glo, Incucyte proliferation assays, metaphase spread analysis and high-content imaging analyses was processed in Microsoft Excel before statistical analysis and visualisation through GraphPad Prism (versions 9 and 10). TIFFs for immunoblotting images were processed using Adobe Photoshop tools Image Rotation, Crop and Scale, with no other tool used. JPEG files for representative microscopy images were processed using Adobe Photoshop tools Crop and Scale, with no other tool used.

For manuscripts utilizing custom algorithms or software that are central to the research but not yet described in published literature, software must be made available to editors and reviewers. We strongly encourage code deposition in a community repository (e.g. GitHub). See the Nature Portfolio guidelines for submitting code & software for further information.

Data

Policy information about availability of data

All manuscripts must include a data availability statement. This statement should provide the following information, where applicable:

- Accession codes, unique identifiers, or web links for publicly available datasets
- A description of any restrictions on data availability
- For clinical datasets or third party data, please ensure that the statement adheres to our policy

Sequencing data from CRISPR screens generated in this study have been deposited in the NCBI GEO Omnibus database. Raw sequencing data from the CRISPR dropout screen (12 files from Illumina HiSeq 4000 single-end 100bp sequencing of Brunello sgRNA libraries obtained from day 6 and day 16 of Cas9 induction in wild type and EXO1 KO cells) and sgRNA counts from MAGeCK analysis have been deposited under accession code GSE255664 [https://www.ncbi.nlm.nih.gov/geo/query/acc.cgi?acc=GSE255664]. Raw sequencing data from CRISPR rescue screens (9 files from Illumina NovaSeq 6000 paired-end 100bp sequencing of Brunello sgRNA libraries from day 10 of Cas9 induction in EXO1 KO +sgNT, EXO1 KO +sgFANCG and EXO1 KO +sgZRSR2 cells) and sgRNA counts from MAGeCK analyses have been deposited under accession code GSE255579 [https://www.ncbi.nlm.nih.gov/geo/query/acc.cgi?acc=GSE255579]. Cancer genomics data used in this study were obtained via cBioPortal, with raw data from 32 TCGA studies and the ICGC/TCGA pan-cancer analysis of whole genomes available for download on their website [https://www.cbioportal.org/datasets]. Source data are provided with this paper.

Research involving human participants, their data, or biological material

Policy information about studies with human participants or human data. See also policy information about sex, gender (identity/presentation), and sexual orientation and race, ethnicity and racism.

Reporting on sex and gender	*Use the terms sex (biological attribute) and gender (shaped by social and cultural circumstances) carefully in order to avoid confusing both terms. Indicate if findings apply to only one sex or gender; describe whether sex and gender were considered in study design; whether sex and/or gender was determined based on self-reporting or assigned and methods used.*<br>*Provide in the source data disaggregated sex and gender data, where this information has been collected, and if consent has been obtained for sharing of individual-level data; provide overall numbers in this Reporting Summary. Please state if this information has not been collected.*<br>*Report sex- and gender-based analyses where performed, justify reasons for lack of sex- and gender-based analysis.*
Reporting on race, ethnicity, or other socially relevant groupings	*Please specify the socially constructed or socially relevant categorization variable(s) used in your manuscript and explain why they were used. Please note that such variables should not be used as proxies for other socially constructed/relevant variables (for example, race or ethnicity should not be used as a proxy for socioeconomic status).*<br>*Provide clear definitions of the relevant terms used, how they were provided (by the participants/respondents, the researchers, or third parties), and the method(s) used to classify people into the different categories (e.g. self-report, census or administrative data, social media data, etc.)*<br>*Please provide details about how you controlled for confounding variables in your analyses.*
Population characteristics	*Describe the covariate-relevant population characteristics of the human research participants (e.g. age, genotypic information, past and current diagnosis and treatment categories). If you filled out the behavioural & social sciences study design questions and have nothing to add here, write "See above."*
Recruitment	*Describe how participants were recruited. Outline any potential self-selection bias or other biases that may be present and how these are likely to impact results.*
Ethics oversight	*Identify the organization(s) that approved the study protocol.*

Note that full information on the approval of the study protocol must also be provided in the manuscript.

Field-specific reporting

Please select the one below that is the best fit for your research. If you are not sure, read the appropriate sections before making your selection.

☒ Life sciences ☐ Behavioural & social sciences ☐ Ecological, evolutionary & environmental sciences

For a reference copy of the document with all sections, see nature.com/documents/nr-reporting-summary-flat.pdf

Life sciences study design

All studies must disclose on these points even when the disclosure is negative.

Sample size	For high content imaging experiments, a minimum of 750 cells were analysed per sample for each biological replicate. For metaphase phenotype scoring, 100 metaphases was analysed per sample for each biological replicate. For DNA fibre analyses, minimum of 300 fibres was analysed per sample for each biological replicate, while 100 fibres was analysed for fork degradation experiments per sample and for each biological replicate.
Data exclusions	No data was excluded from this study.
Replication	All KO clones generated in this study were functionally validated to ensure that phenotypes correspond to previously published phenotypes.

Replication

Validation of synthetic lethal interactions in eHAP iCas9 cell line was performed with three independent assays: clonogenic assay, Cell Titre Glo assay and Incucyte proliferation assay. Validation of synthetic lethal findings was also performed also in HeLa Kyoto iCas9 EXO1 KO cell line (different origin from eHAP) and with KO generated using different sgRNA. EXO1-FANCG synthetic lethal interaction was also validated with a reciprocal approach using FANCC-deficient PL11 cell line. Replication stress increase in EXO1-FANCG DKO cells prior to cell death was a phenotype observed both by immunofluorescence and imaging of RPA-pS33 foci, as well as by EM analysis of replication forks with observation of gaps. Fanconi Anaemia-deficient phenotype of ZRSR2 KO cells was investigated using canonical FA functionality assay with metaphase spread analysis after MMC treatment, with FANCD2-Ub assay and with investigation of sensitivities to PARPi and cisplatin. Additive sensitivities for exemplar synthetic lethal interactions were performed with multiple PARP inhibitors and in two different cell lines. Phenotype of replication fork speed was reproduced by two different researchers (MM and PK) across 9 biological replicates.

Randomization

Randomization was not relevant for this study.

Blinding

Blinding was not performed in this study. However, in several instances data acquisition and analysis was automated (such as in imaging analyses) to avoid bias, and occasionally experiments were performed by with multiple researchers handling different parts of experiment (metaphase spreads were stained and analysed by TT, with cell culture done by MM; for DNA fibre analysis in Pemetrexed and PARPi experiments PK did the analysis post image acquisition).

Reporting for specific materials, systems and methods

We require information from authors about some types of materials, experimental systems and methods used in many studies. Here, indicate whether each material, system or method listed is relevant to your study. If you are not sure if a list item applies to your research, read the appropriate section before selecting a response.

Materials & experimental systems

n/a	Involved in the study
☐	☒ Antibodies
☐	☒ Eukaryotic cell lines
☒	☐ Palaeontology and archaeology
☒	☐ Animals and other organisms
☒	☐ Clinical data
☒	☐ Dual use research of concern
☒	☐ Plants

Methods

n/a	Involved in the study
☒	☐ ChIP-seq
☐	☒ Flow cytometry
☒	☐ MRI-based neuroimaging

Antibodies

Antibodies used

Commercially available antibodies used in this study: anti-EXO1 (ab95068, Abcam, rabbit, 1:1000), anti-alpha-tubulin (clone B-5-1-2, T6074, Sigma, mouse, 1:10000), anti-vinculin (clone hVIN-1, ab11194, Abcam, mouse, 1:20000), anti-FANCG (clone F-8, sc-393382, Santa Cruz, mouse, 1:1000), anti-KAP1-pS824 (ab70369, Abcam, rabbit, 1:1000), anti-KAP1 (clone 20C1, ab22553, Abcam, mouse, 1:1000), anti-p53-pS15 (9284S, CST, rabbit, 1:1000), anti-p53 (clone 1C12, 2524S, CST, mouse, 1:1000), anti-CHK2-pT68 (2661S, CST, rabbit, 1:1000), anti-CHK2 (clone 7, 05-649, Millipore, mouse, 1:500), anti-H2AX-pS139 (gamma-H2AX, clone JBW301, 05-636, Millipore, mouse, 1:1000), anti-histone H3 (ab1791, Abcam, rabbit, 1:5000), anti-RPA-pS33 (A300-246A, Bethyl Laboratories, rabbit, 1:1000), anti-RPA (clone 9H8, ab2175, Abcam, mouse, 1:1000), anti-FANCD2 (clone EPR2302, ab108928, Abcam, rabbit, 1:1000), anti-RMI2 (ab122685, Abcam, rabbit, 1:500), anti-FAM175A/Abraxas1 (clone EPR6310(2), ab139191, Abcam, rabbit, 1:1000), anti-BRCC36 (A302-517A-M, Bethyl Laboratories, rabbit, 1:1000), anti-FANCC (clone 8F3, MABC524, Sigma, mouse, 1:1000), anti-BrdU (ab6326, Abcam), anti-BrdU (clone B44, 347580, Becton Dickinson). Anti-FAAP24 antibody was a kind gift from Stephen West (rabbit, 1:1000) and anti-APITD1/MHF1 antibody was a kind gift from Weidong Wang (rabbit, 1:1000).

Validation

Validation of commercially available antibodies for specific application used in this study was performed as described by the manufacturer and as described in literature (cited through manufacturer's website and through CiteAb reagent search engine). Anti-BRCC36 (A302-517A-M, Bethyl Laboratories) was validated in this study for immunoblotting through specific depletion via sgRNA. Non-commercially available antibodies used in this study were validated as described in the original publication and in this study through specific depletion via sgRNA: anti-FAAP24 (PMID: 17289582) and anti-APITD1/MHF1 (PMID: 20347428).

Eukaryotic cell lines

Policy information about cell lines and Sex and Gender in Research

Cell line source(s)

This study used cancer cell lines eHAP (derived from a male patient), HeLa Kyoto and PL11 (both derived from female patients). eHAP cell line is a diploidised HAP1 cell line, derived from chronic myeloid leukemia KBM-7 cell line (haploid originally purchased from Horizon Discovery). HeLa Kyoto cell line was derived from human papillomavirus-related cervical adenocarcinoma HeLa cell line. PL11 cell line (Panc 03.27) was derived from a pancreatic adenocarcinoma patient and obtained via Alan D'Andrea lab (DFCI, US).

Authentication

eHAP cell line was karyotyped by low pass sequencing at The Francis Crick Institute, while other cell lines have not been authenticated for this particular study.

Mycoplasma contamination

All cell lines used in this study have been regularly tested for mycoplasma contamination by Cell Services facility at The

Mycoplasma contamination | Francis Crick Institute (using PCR, imaging and soft agar methods). None of the cell lines used in the study had tested positive for mycoplasma contamination.

Commonly misidentified lines (See ICLAC register) | *Name any commonly misidentified cell lines used in the study and provide a rationale for their use.*

Plants

Seed stocks | *Report on the source of all seed stocks or other plant material used. If applicable, state the seed stock centre and catalogue number. If plant specimens were collected from the field, describe the collection location, date and sampling procedures.*

Novel plant genotypes | *Describe the methods by which all novel plant genotypes were produced. This includes those generated by transgenic approaches, gene editing, chemical/radiation-based mutagenesis and hybridization. For transgenic lines, describe the transformation method, the number of independent lines analyzed and the generation upon which experiments were performed. For gene-edited lines, describe the editor used, the endogenous sequence targeted for editing, the targeting guide RNA sequence (if applicable) and how the editor was applied.*

Authentication | *Describe any authentication procedures for each seed stock used or novel genotype generated. Describe any experiments used to assess the effect of a mutation and, where applicable, how potential secondary effects (e.g. second site T-DNA insertions, mosiacism, off-target gene editing) were examined.*

Flow Cytometry

Plots

Confirm that:

☒ The axis labels state the marker and fluorochrome used (e.g. CD4-FITC).

☒ The axis scales are clearly visible. Include numbers along axes only for bottom left plot of group (a 'group' is an analysis of identical markers).

☒ All plots are contour plots with outliers or pseudocolor plots.

☒ A numerical value for number of cells or percentage (with statistics) is provided.

Methodology

Sample preparation | For Cas9 cutting efficiency analyses, cells were harvested by trypsinising, pelleted, resuspended in 1x PBS and kept on ice in dark prior to analysis. For cell cycle analyses (as described in detail in Methods) cells were incubated with EdU and fixed with PFA. Fixed cells were then used for Click-iT reaction (Alexa fluor 488) for EdU and were stained with DAPI prior to analyses.

Instrument | BD LSRFortessa Cell Analyzer was used for signal acquisition.

Software | BD FACSDiva software was used for data acquisition, while FlowJo software was used for gating.

Cell population abundance | For Cas9 cutting efficiency analysis, 20000 cells were analysed as BFP+. For cell cycle analyses, 10000 cells were analysed as 'Single cells'. No sorting of specific cell populations was included in flow cytometry experiments included within this manuscript.

Gating strategy | Cells were gated using Forward Scatter Area (FSC-A) vs Side Scatter Area (SSC-A) to remove debris. 'Single cells' were then gated from doublets/clumps using FSC-H vs FSC-A ratio and gating of objects with FSC-A:FSC-H ratio of ~1. For Cas9 cutting efficiency analysis, BFP+ population was gated (450-50-A vs FSC-A) and then the BFP+/GFP- population was gated with the use of a non-Dox control (450-50-A vs 530-30-A). For cell cycle analyses, 'Single cells' were gated with a gating strategy as shown in Extended Data Figure 5g (530-30-A vs 450-50-A).

☒ Tick this box to confirm that a figure exemplifying the gating strategy is provided in the Supplementary Information.
